# Removal of Malachite Green Dye from Aqueous Solution by Catalytic Wet Oxidation Technique Using Ni/Kaolin as Catalyst

**DOI:** 10.3390/molecules27217528

**Published:** 2022-11-03

**Authors:** Abdelhak Moumen, Youghourta Belhocine, Najoua Sbei, Seyfeddine Rahali, Fatima Adam Mohamed Ali, Fedia Mechati, Fouad Hamdaoui, Mahamadou Seydou

**Affiliations:** 1 Department of Petrochemical, Faculty of Technology, 20 August 1955 University of Skikda, El Hadaik Road, Skikda 21000, Algeria; 2 Laboratory of Catalysis, Bioprocess and Environment, Department of Process Engineering, Faculty of Technology, 20 August 1955 University of Skikda, El Hadaik Road, Skikda 21000, Algeria; 3 Institute of Nanotechnology, Karlsruhe Institute of Technology, Eggenstein Leopoldshafen, 76344 Karlsruhe, Germany; 4 Department of Chemistry, College of Science and Arts, Qassim University, Ar Rass, Saudi Arabia; 5 Department of Chemistry, College of Science, Imam Mohammad Ibn Saud Islamic University (IMSIU), Riyadh 11432, Saudi Arabia; 6 ITODYS, CNRS, UMR 7086, University de Paris, 15 rue J.A. de Baïf, 75013 Paris, France

**Keywords:** kaolin, malachite green, nickel oxide, catalyst, impregnation, wet hydrogen peroxide catalytic oxidation

## Abstract

In this study, natural Algerian kaolin was used as a support and impregnated with nickel at different loading amounts (2 wt.%, 5 wt.%, and 7 wt.%) in order to prepare a supported catalyst. The wet impregnation technique was used in this preparation; nickel oxide (NiO) was the active phase precursor of the catalyst, and the catalysts were designated as follows: 2%, 5%, and 7% Ni/kaolin. These catalysts were put to the test in catalytic wet peroxide oxidation (CWPO) for degrading the organic contaminant malachite green dye (MG). Analytical techniques such as FTIR spectroscopy, X-ray diffraction, BET, and X-fluorescence were used to examine the structure, morphology, and chemical composition of the support and the produced catalysts. Several parameters, including temperature, catalytic dose, metal loading, hydrogen peroxide volume, and kinetic model were systematically investigated. The combination of improved parameters resulted in a significant increase in the catalytic activity, achieving a high removal rate of MG dye of 98.87%.

## 1. Introduction

Due to the high concentration of pollutants they contain, aqueous effluents polluted by organic matter such as dyes from a chemical activity are very few biodegradable [1]. Malachite green (MG) dye is a commonly used colorant in a variety of industries, including textiles, paper, and leather [2]. However, it has been demonstrated that MG is toxic to freshwater animals in both acute and chronic exposures [3], and it is particularly toxic to mammalian organs such as the liver, kidney, and skin [4,5]. As a result, it is critical to eliminate dyestuff wastewater from the environment [6]. To achieve this goal, many scientists explored different strategies for dye removal based on physical, chemical, and biological processes [7]. Filtration, precipitation, flocculation, adsorption, and ion exchange are only a few examples [8,9,10,11,12,13].

Because it is inexpensive, adaptable, and easy, the adsorption method [14,15] is one of the most preferred processes. This is in contrast to most other methods, which are ineffectual due to the cost or are ineffective at high and/or low contamination concentrations [14]. Adsorption has the advantage of being able to be used at extremely low concentrations, as well as the ability to regenerate and reuse the adsorbent [16]. To effectively remove pollutants, several types of advanced oxidation techniques have been used, such as the wet air oxidation process (WAO); this requires very high temperatures and pressures, resulting in high installation costs, and therefore the practical uses of this process are limited [17,18]. To avoid these disadvantages, research efforts in this field have primarily focused on cost-effective and environmentally friendly alternatives, such as catalytic wet peroxide oxidation (CWPO), where hydrogen peroxide is used as an oxidant. This process produces free hydroxyl radicals (OH•), powerful oxidants of organic pollutants [19,20] which are cost-effective in decomposing the most complex contaminants [21] to inorganic compounds such as CO_2_ and H_2_O. The most notable benefit of this technique is the widespread use of heterogeneous catalysts, such as supported catalysts for water treatment [16], to reduce the severity of the oxidation conditions [17,18].

Kaolin clay is a non–toxic material that has been the subject of substantial research in the field of clays and soils due to its potential use as catalyst support. Kaolin was used as a support in the manufacture of supported catalysts in numerous studies conducted all over the world [16,20,22,23,24,25]. These catalysts have been utilized in wastewater treatment to degrade organic pollutants [20,26].

Due to their availability, low cost, and chemical qualities, transition metals such as cobalt, manganese, iron, copper, nickel, etc., and metal oxides [27,28,29,30,31,32] are the most widely utilized in the fabrication of catalysts. However, transition metal ions are dangerous and difficult to recycle, and therefore their application in wastewater treatment systems is restricted [16,33]. On the other hand, because of its high specific capacity, inherent environmental friendliness, low cost, and high efficiency, nickel oxide (NiO) has gained a lot of attention among metal oxides [34]. Nickel oxide (NiO) particles are highly frequent in catalyst coatings due to their ease of accessibility.

In this work, we have developed a supported catalyst by impregnating nickel on local natural kaolin as support. The catalytic activity against the MG dye in water [35] was evaluated by considering experimental variables such as the temperature, the oxidant amount, and the catalyst mass.

## 2. Experimental

### 2.1. Materials and Methods

#### Materials

The kaolin used in this work is collected in the region of Tamazert (North-East of Algeria) and procured from the sanitary ceramic company of El-Milia, Algeria.

**Figure 1:**
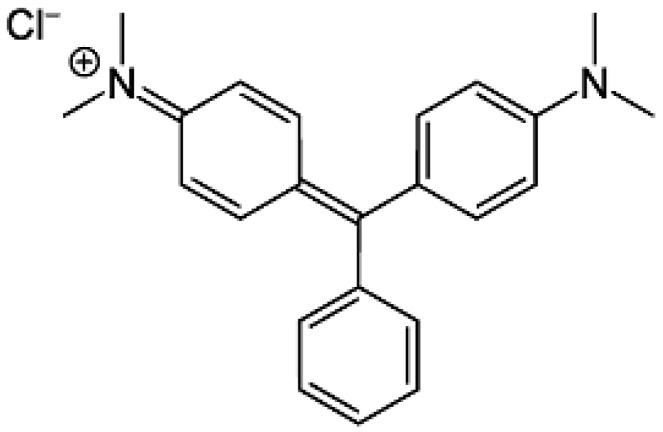
Chemical structure of MG dye.

All the chemicals employed in the present study were of analytical reagent grade. Hydrogen peroxide (H_2_O_2_), caustic soda (NaOH), nickel (II) nitrate hexahydrate Ni(NO_3_)_2_∙6H_2_O, and malachite green (MG) were purchased from Merck Chemical Company (Darmstadt, Germany). The chemical structure of the MG dye is displayed in Figure 1.

### 2.2. Synthesis of the Supported Catalysts

The supported catalysts were synthesized according to the following experimental protocol: the wet impregnation method was used to produce tailored catalysts with good dispersion of the active phase. To prepare a sufficient amount of catalyst, we started with a known mass of catalyst; from this, we calculated the mass of the kaolin support, which had been prepared beforehand, and the mass of metal impregnated with the desired loading amounts (2%, 5%, and 7%). The support (kaolin) was previously washed in distilled water and dried at 100 °C. A known concentration of the aqueous solution of precursor Ni(NO_3_)_2_∙6H_2_O was prepared. After that, this was added to the support (kaolin), which was first wetted in a volume of water. Complete nitrate precipitation was ensured by gradually adding NaOH until a pH of 10 was reached. Then, the mixture was put under magnetic stirring at room temperature for 3 h; at the end of stirring, the mixture underwent several treatments. To evaporate the solvents existing in the mixture, we used a sand bath at a constant temperature for 5 h.

At the end, after filtration, we used two heat treatments: drying and calcination. The solid obtained was dried in the oven for 24 h at a temperature of 80 °C, then brayed and calcined at 400 °C for 3 h. The same process was used for all of the samples (2% Ni/kaolin, 5% Ni/kaolin, and 7% Ni/kaolin).

#### 2.2.1. Apparatus

The mineral and elemental composition of the kaolin sample was performed by X-Ray Fluorescence (XRF) analysis using a Siemens-type apparatus (SRS 3000, Munich, Germany). The crystalline structure of the samples was identified by X-Ray diffraction (XRD) analysis using PANalyticalX’Pert PRO diffractometer (Malvern, UK) with Cu/K_α_ radiation (λ = 0.1540 nm) and scanning over the 2θ range from 10 to 90°. The infrared spectra were recorded on a Thermo Scientific Nicolet iS10 Fourier transform spectrometer in the spectral range between 4000 and 400 cm^−1^. BET analysis was performed using nitrogen gas adsorption/desorption isotherms obtained at 77 K with a Micromeritics Accusorb 2100E (Norcross, GA, USA) model surface area analyzer. The porosity of the kaolin was also examined. A UV-vis spectrometer (OPTIZEN 3220UV, Warsawa, Poland) was used to measure the concentration of MG in the sample at 617 nm.

#### 2.2.2. Catalytic Activity Test

The catalytic activity of the Ni/kaolin catalyst was tested in the catalytic oxidation of malachite green dye using H_2_O_2_ (30%) as an oxidant reagent at the flow rate. All the samples were prepared and distributed in 250 mL glass breakers, into which 200 mL of the solution of MG with C = 20 mg/L and 0.2 g of the catalyst for different amounts (2 wt.%, 5 wt.%, and 7 wt.%) were added. The reaction was kept going for 3 h with constant stirring at 320 rpm. Samples were collected every 15 min and analyzed by UV-Vis spectroscopy.

The percentage removal of dye was calculated using the following equation:

(1) $$Dye\;removal\;\left(\%\right)=\frac{C_{0}-C_{t\;}}{C_{0}}\times 100$$

where *C*_0_ and *C_t_* are the concentrations of dye in solution (mg/L) at reaction times 0 and *t*, respectively. The experimental data are fit using a pseudo-first-order kinetics model defined by the Matthews–Weber equation, where *k* (min^−1^) is the reaction rate constant.

(2) $$\ln\frac{C_{t}}{C_{0}}=-kt$$

The effect of catalyst dose, oxidant volume, and the temperature was investigated.

## 3. Results and Discussion

### 3.1. Support and Catalyst Characterization

#### 3.1.1. The Chemical Composition of Support (Natural Kaolin)

The chemical analysis of the kaolin is shown in Table 1; the kaolin is relatively rich in silica (43.82%) and alumina (36.66%), and it also contains a low quantity of oxides. The loss on ignition of pure kaolin can be estimated theoretically from the molecular mass of kaolinite. The loss of a mass of pure kaolin is evaluated at 16.36%.

#### 3.1.2. Specific Surface Area (SBET) and Pore Size

**Figure 2:**
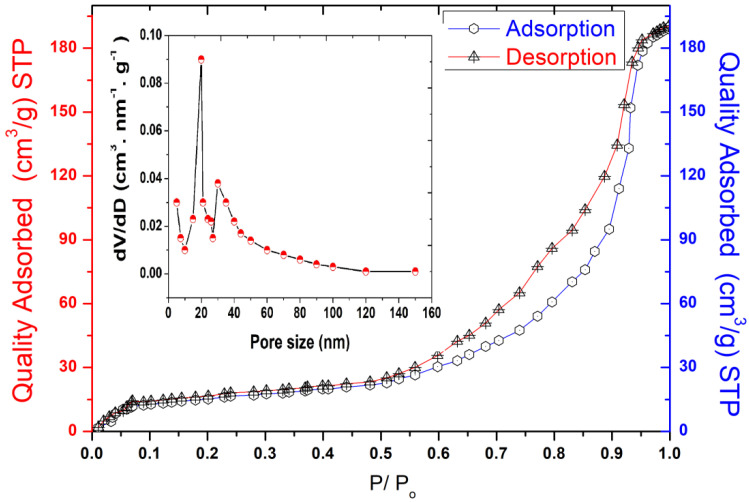
Size distribution analysis, BET adsorption, and desorption isotherms of Tamazert kaolin.

The pore size, specific surface area (SBET), and micropore volume are considered key parameters in evaluating the adsorption capacity of materials. The SBET of the kaolin was studied using the nitrogen gas adsorption and desorption isotherms, as shown in Figure 2.

The obtained results show that the BET curve fits the type IV isotherm according to the classification of the IPUAC, with an H3 hysteresis loop ranging between 0.50 and 0.90 relative pressure (P/P_0_), indicating that this support has a typical mesoporous structure enhanced by the presence of meso and macroporous morphologies on its surface. The specific surface area of Tamazert kaolin was found to be 31.2 m^2^ g^−1^, and the pore size distribution was reported to be in the range of 5–15 nm, as shown in Figure 2.

#### 3.1.3. FT-IR Spectroscopic Analysis

**Figure 3:**
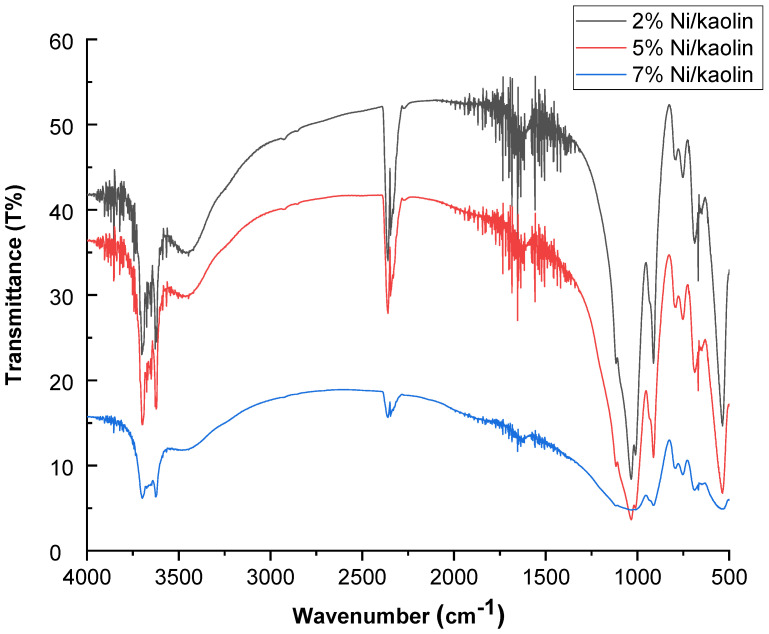
FT-IR spectra of supported catalysts (2%, 5%, and 7% Ni/kaolin).

FT-IR spectra for 2%, 5%, and 7% Ni/kaolin are represented in Figure 3.

Two intense absorption bands are located at 3600 and 3690 cm^−1^, which are generally assigned to hydroxyl groups (OH^−^). The band at 1620 cm^−1^ corresponds to the vibration of water molecules. Si–O stretching vibrations appeared at 1119 cm^−1^, while the asymmetrical Si–O–Si stretching vibrations occurred around 1032 cm^−1^. Absorption bands at 475 cm^−1^ can be attributed to the stretching vibration of the Ni–O bond [24].

#### 3.1.4. XRD Analysis

**Figure 4:**
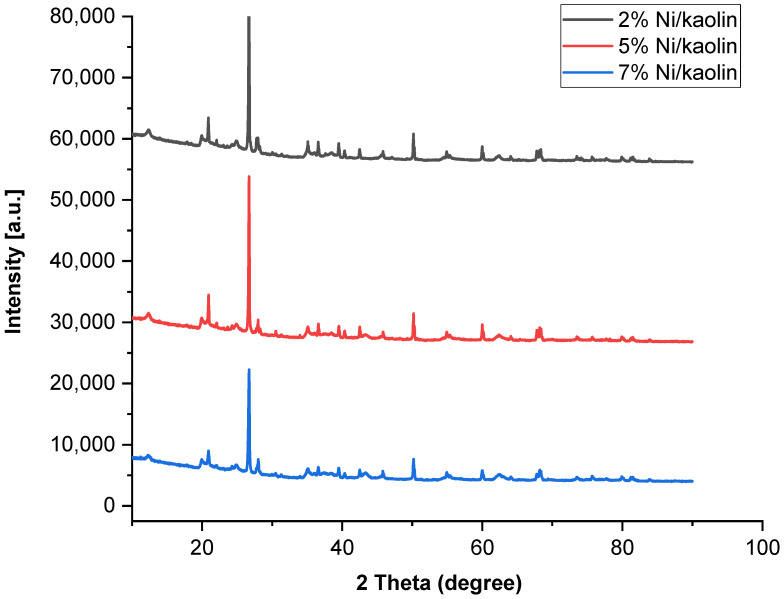
X-ray diffractograms of supported catalysts (2%, 5%, and 7% of Ni/kaolin).

Structural modifications occurring in the treated kaolin were studied using the X-ray diffraction technique. The XRD patterns were recorded in the range of 10–90°. Figure 4 shows the XRD profiles of catalysts (2%, 5%, and 7% Ni/kaolin).

The raw kaolin exhibits narrow peaks, indicating the existence of crystalline quartz phases (2θ = 12, 20, 22, 25, and 56°). In the supported catalysts, the presence of peaks at 2θ = 37.3, 43.3, 62.8, and 75.3° could indicate the presence of NiO at the surface of the kaolin according to JCPDS 47-1049 [36]. As shown in Figure 4, the main peaks have low intensity due to low Ni loading. Jiang et al. [37] obtained similar results. The good crystallinity of the catalyst is confirmed by the narrowness of the peaks.

## 4. Effect of the Experimental Parameters

The examination of the catalytic characteristics of the prepared catalysts in the presence of hydrogen peroxide was performed using different parameters such as the catalyst dose, the amount of H_2_O_2_, the metal loading of the catalyst and the temperature.

### 4.1. Effect of Metal Loading

**Figure 5:**
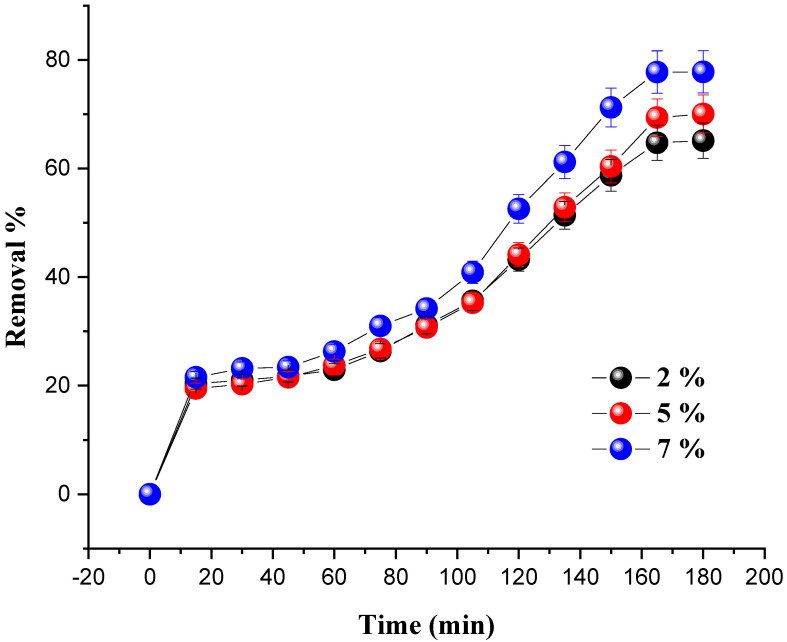
Effect of nickel loading on MG removal in terms of time. (Reaction conditions: *C*_0_ = 20 mg/L, m = 0.2 g, V_H__2O__2_ = 10 mL, pH = 7 and T = 25 °C).

To elucidate the effect of metal loading on the catalytic performance of the prepared catalysts, different amounts of Ni/kaolin (2 wt.%, 5 wt.%, and 7 wt.%) were loaded at varied time intervals in order for the dye to reach its removal percentage by keeping a constant weight of the catalyst (0.2 g), while progressively increasing the contact time. Every 15 min, a small sample of the reaction mixture was centrifuged for 10 min before the absorbance was measured with a UV-Vis spectrophotometer. The influence of the percentage of nickel loading on dye removal is shown in Figure 5.

The results revealed that nickel loading has an impact on the degradation of MG. Indeed, when the amount of nickel in the solution was increased from 2 to 7 wt.%, the decolorization of malachite green gradually increased until it reached its maximum value, leading to an increase in the generation of (OH•) radicals in the system. A rate of 77.78% was achieved for 7 wt.% of Ni/kaolin loading within 180 min; therefore, this optimal percentage (7 wt.% of Ni/kaolin) was used as a reference in the subsequent investigations.

### 4.2. Effect of the Catalyst Mass

**Figure 6:**
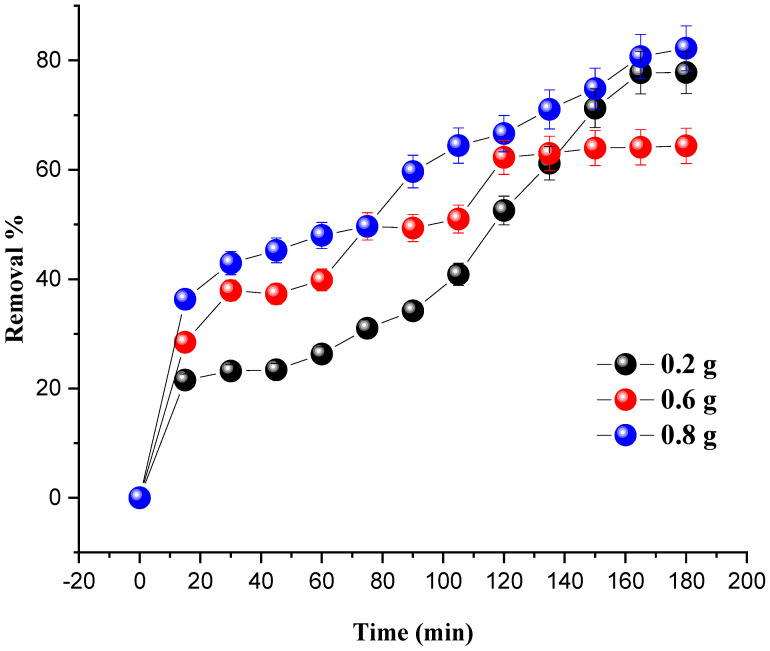
Effect of catalyst loading on MG removal. Reaction conditions: *C*_0_ = 20 mg/L, V_H2O2_ = 10 mL, pH = 7 and T = 25 °C.

The amount of MG degraded at room temperature under the influence of the catalyst mass is shown in Figure 6. The mass of the catalyst (7% Ni/kaolin) varied from 0.2 to 0.8 g, and the initial concentration of the pollutant was set at 20 mg/L.

The results from Figure 6 show that the degradation of MG is proportional to the dose of the catalyst. An increase in catalyst dose leads to an improvement in the number of active sites responsible for the catalytic activity, therefore increasing the percentage degradation of the dye. The optimum dye degradation rate (82.20%) was achieved at a catalyst mass of 0.8 g.

### 4.3. Effect of Oxidant Amount

**Figure 7:**
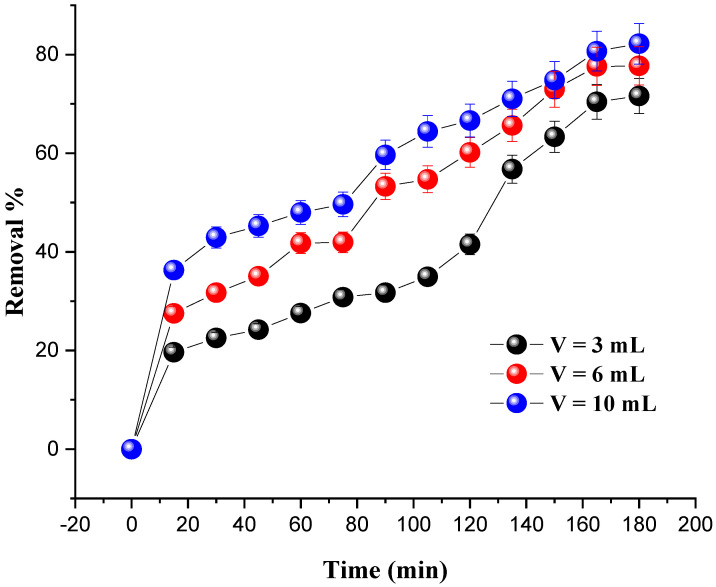
Effect of oxidant volume on MG removal. Reaction conditions: C_0_ = 20 mg/L, m = 0.8 g, pH = 7 and T = 25 °C.

The effect of H_2_O_2_ dosage on MG degradation was assessed by adding several H_2_O_2_ volumes (3 mL, 6 mL, and 10 mL) under the following conditions: the amount of catalyst was set at 0.8 g and the dye concentration at 20 mg/L; the pH of the solutions was set at 7 and the temperature at 25 °C. The results are presented in Figure 7.

It was found that the degradation rate of the dye was significantly increased with the increase in the amount of hydrogen peroxide. The optimum degradation rate (82.20%) was obtained for an oxidant amount of 10 mL after 180 min. The excess of H_2_O_2_ and the presence of a catalyst promote the formation of large numbers of (OH•) radicals, which are responsible for the degradation of the dye due to their potential to convert the molecules of MG into intermediate products.

### 4.4. Effect of Temperature

The influence of the temperature was studied in the temperature range of 25–50 °C and under the following operating conditions: a mass of catalyst of 0.8 g; a dye concentration of 20 mg/L; and 10 mL of H_2_O_2_. To keep the temperature at the target value, a stirring plate and a thermometer were used.

**Figure 8:**
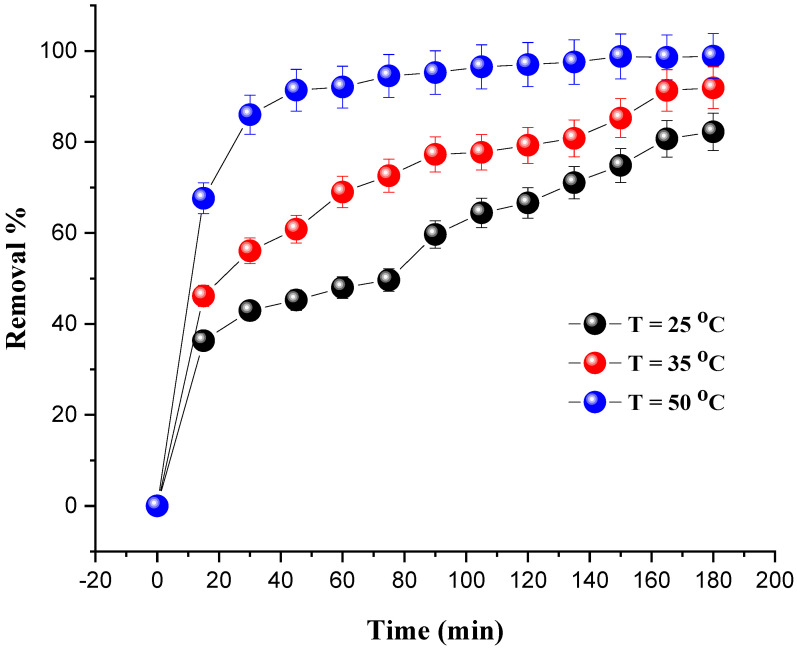
Effect of temperature on MG removal. Reaction conditions: *C*_0_ = 20 mg/L, V_H2O2_ = 10 mL, pH = 7 and m = 0.8 g.

Figure 8 shows the percentage increase in the degradation of MG with the evolution of the temperature. We notice that the optimal degradation rate (98.87%) was obtained at 50 °C after 180 min. On the other hand, as the temperature increases, exothermic adsorption of the reactants becomes less desirable due to a decrease in the apparent activation energy. Additionally, the increase in temperature leads to an increase in the rate of reaction between H_2_O_2_ and the catalyst, and thus increases the rate of generation of oxidizing species, such as hydroxyl free radicals that are efficient to degrade the dye [38]. The same conclusions regarding the temperature effect were drawn in previous studies [39,40,41,42,43].

## 5. Kinetic Study of MG Removal during the Oxidation Process

**Figure 9:**
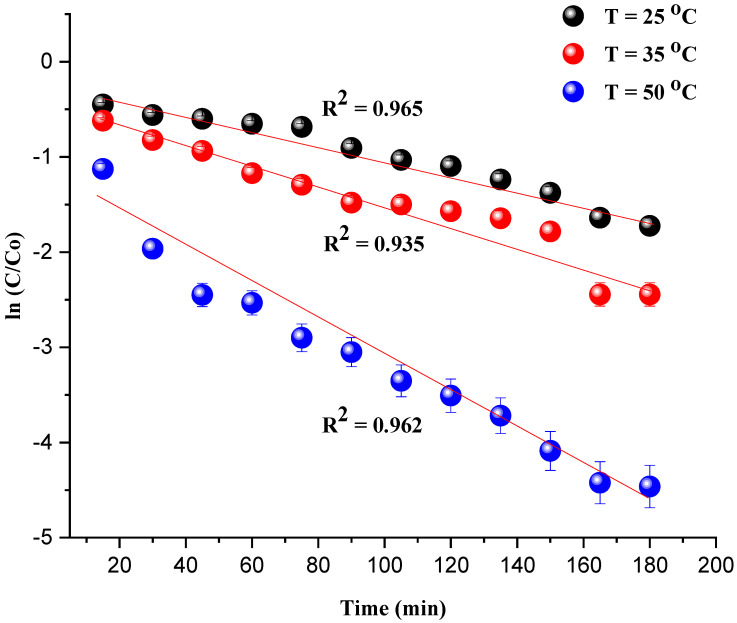
ln(*C*/*C*_0_) as a function of time *t* (min) (Reaction conditions: *C*_0_ = 20 mg/L, catalyst mass = 0.8 g, and pH = 7).

The kinetic investigation of the degradation of MG is essential to evaluate the catalytic performance of the catalyst Ni/kaolin in the presence of H_2_O_2_. The straightline plot of Figure 9 showed that the degradation of the MG undergoes a first-order pseudo-reaction. The rate constant value (*k*) was determined from the slope of the plot.

The coefficient of determination R^2^ and apparent rate constants *k_app_* at different temperatures are presented in Table 2. It should be noted that the observed deviation of the last two data points from the linear trend for the plot of ln(*C*/*C*_0_) vs. *T* (35 °C) can be attributed to the interaction of the intermediate products of the degradation with the remaining MG dye.

Based on the Arrhenius formula, the activation energy of the degradation of MG by the catalyst Ni/kaolin in the presence of hydrogen peroxide was calculated according to Equation (3):

(3) $$\ln k_{app}=\ln k_{0}-\frac{E_{a}}{RT}$$

where *k*_0_ is the pre-exponential factor, and *E_a_* denotes the apparent activation energy kJ/mol.

**Figure 10:**
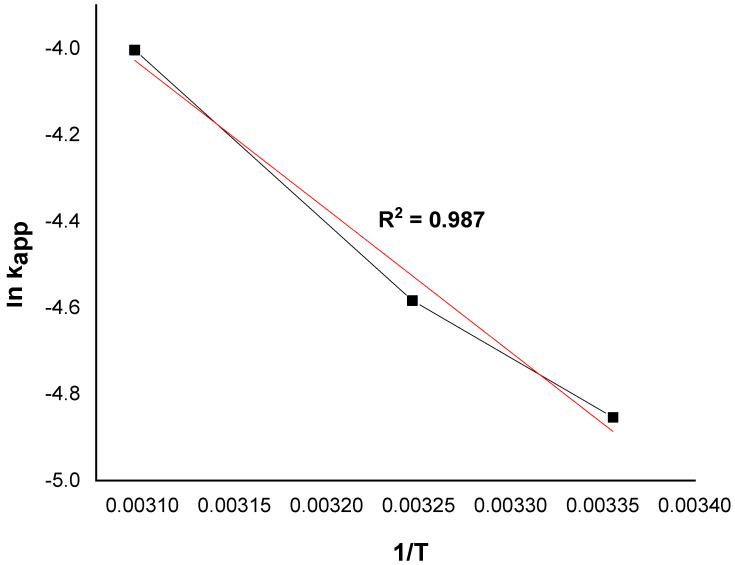
The plot of ln*k_app_* versus 1/*T* at different temperatures.

The value of the activation energy was calculated from the regression slope (line of ln*k_app_* as a function of 1/*T*), as illustrated in Figure 10.

The apparent activation energy of the catalytic oxidation of the dye is 27.51 kJ/mol. Furthermore, the typical activation energy *E_a_* ranges between 60 and 250 kJ/mol [44,45]. The reported value is quite similar to that of organic pollutants degraded by hydroxyl radicals (≈30 kJ/mol) [46]. The low value of the activation energy suggests that the degradation of MG is limited by a diffusion step, and the apparent constant rate reflects the rate at which the MG molecules migrate from the solution to the catalyst surface where the reaction takes place. A similar result was obtained by Behnajady et al. [47]. On the other hand, any increase in temperature can provide more energy for the reactive molecules to dominate the activation energy of the reaction [38]. This result indicates that Ni/kaolin catalyst faces a low energy barrier and can be activated easily in the presence of H_2_O_2_ [48].

## 6. Comparative Investigation of MG Removal on Different Catalysts

Due to the different experimental conditions used in prior investigations, a direct comparison of the adsorption capabilities investigated in this work with those previously published in the literature is challenging. However, an examination of Table 3 shows that Ni/kaolin catalyst used in this study exhibits a better MG removal rate than other catalysts [49,50,51,52], as listed in Table 3.

## 7. Degradation Mechanism of MG Dye

The mechanism for the decomposition of H_2_O_2_ molecules and the production of reactive species in the NiO/kaolin/H_2_O_2_ system is proposed in this article. The OH• radicals are produced by heterogenous reactions that occur in the solid phase between the adsorption of H_2_O_2_ molecules and the actives sites of the catalyst, according to Equation (4), leading to the generation of OH• (_ads_). It has to be noted that the adsorption of the dye (Equation (5)) and H_2_O_2_ molecules on the catalyst surface facilitates the reaction between the oxidant and the active sites to produce free radicals in the solid phase [22]. Finally, the OH• (_ads_) radicals directly attack the dye molecule (MG) to obtain degraded products (Equation (6)).

Catalyst + H_2_O_2_ → catalyst (OH•)_ads_ (adsorption of H_2_O_2_ on the surface of catalyst) (4)

Dye + catalyst → catalyst (dye)_ads_ (adsorption of the dye on the surface of catalyst (5)

OH• + organic toxic dye (MG) → degraded products(6)

## 8. Conclusions

In this study, the catalytic activity of the supported catalyst, which was prepared by the impregnation technique (kaolin impregnated by nickel) on the degradation of the malachite green dye in the presence of hydrogen peroxide H_2_O_2,_ was investigated. The experimental tests carried out allowed us to determine the optimal reaction conditions for the degradation of MG, which are the percentage of nickel 7%, the reaction temperature of 50 °C, the mass of catalyst 0.8 g, the volume of H_2_O_2_ 10 mL, and the pH = 7. Under these conditions, the removal rate of malachite green dye reached a maximum of 98.87% after 3 h. The increase in Ni content in the catalyst increases and improves the degradation efficiency of the malachite green. The kinetic study showed that the degradation reaction of the malachite green follows the pseudo-first-order. The results of this study evidenced the feasibility and the effectiveness of supported catalysts prepared by the wet impregnation method as potential and cost-effective adsorbents for the removal of pollutant dyes.

## Figures and Tables

**Table 1:** The chemical composition (wt.%) of natural kaolin (derived from XRF analysis).

Oxides	SiO_2_	Fe_2_O_3_	Al_2_O_3_	CaCO_3_	CaO	MgO	MnO	PAF
(wt.%)	43.82	0.55	36.66	1.35	1.05	0.05	0.1	16.36

**Table 2:** The coefficient of determination R^2^ and apparent rate constants *k_app_*_._

Temperature	*k_app_*	R^2^	σ (Standard Deviation)
T = 298 K	0.0078	0.965	0.495
T = 308 K	0.0102	0.936	0.683
T = 323 K	0.0182	0.962	1.297

**Table 3:** Comparison of the percentage degradation of MG dye by supported catalysts reported in the literature.

Catalyst	Degradation %	References
Ag/ZnCo-ZIF	98%	[49]
NiO/Al_2_O_3_	82%	[50]
Chitosan/ZnO	54%	[51]
Chitosan/Ce-ZnO	87%	[51]
Mn-ZnO/CNT	95%	[52]
Ni/kaolin	98.87%	This work

## Data Availability

The data presented in this study are available on reasonable request from the corresponding author.

## References

[1] Lahbabi N., Rais Z., Hajjaji M., Kacim S. (2009). Oxydation du phenol sur un catalyseur à base de Fer supporté sur une argile marocaine. Afr. Sci..

[2] Forgacs E., Cserhati T., Oros G. (2004). Removal of synthetic dyes from wastewaters: A review. Environ. Int..

[3] Bayramoglu G., Arica M.Y. (2013). Removal of reactive dyes from wastewater by acrylate polymer beads bearing amino groups: Isotherm and kinetic studies. Color. Technol..

[4] Zhang J., Li Y., Zhang C., Jing Y. (2008). Adsorption of malachite green from aqueous solution onto carbon prepared from Arundo donax root. J. Hazard. Mater..

[5] Yonar M.E., Yonar S.M. (2010). Changes in selected immunological parameters and antioxidant status of rainbow trout exposed to malachite green (Oncorhynchus mykiss, Walbaum, 1792). Pestic. Biochem. Physiol..

[6] Panandiker A., Fernandes C., Rao T.K.G., Rao K.V.K. (1993). Morphological transformation of Syrian hamster embryo cells in primary culture by Malachite Green correlates well with the evidence for formation of reactive free radicals. Cancer Lett..

[7] Zhou L., Gao C., Xu W. (2010). Magnetic Dendritic Materials for Highly Efficient Adsorption of Dyes and Drugs. ACS Appl. Mater. Interfaces.

[8] Liu F., Chung S., Oh G., Seo T.S. (2012). Three-dimensional graphene oxide nanostructure for fast and efficient water-soluble dye removal. ACS Appl. Mater. Interfaces.

[9] Khosa M.A., Shah S.S., Feng X. (2014). Metal sericin complexation and ultrafiltration of heavy metals from aqueous solution. Chem. Eng. J..

[10] Jung C., Phal N., Oh J., Chu K.H., Jang M., Yoon Y. (2015). Removal of humic and tannic acids by adsorption-coagulation combined systems with activated biochar. J. Hazard. Mater..

[11] Pu Y., Yang X., Hong Z., Wang D., Yu S., Jie H. (2013). Adsorption and desorption of thallium(I) on multiwalled carbon nanotubes. Chem. Eng. J..

[12] Guo H.M., Yang L.J., Zhou X.Q. (2014). Simultaneous Removal of Fluoride and Arsenic from Aqueous Solution using Activated Red Mud. Sep. Sci. Technol..

[13] Chan B., Dudeney A. (2008). Reverse osmosis removal of arsenic residues from bioleaching of refractory gold concentrates. Miner. Eng..

[14] Tran H.H., Roddick F.A., O’Donnell J.A. (1999). Comparison of Chromatography and Desiccant Silica Gels for the Adsorption of Metal Ions—I. Adsorption and Kinetics. Water Res..

[15] Mohanty K., Das D., Biswas M.N. (2006). Preparation and characterization of activated carbons from Sterculia alata nutshell by chemical activation with zinc chloride to remove phenol from wastewater. Adsorption.

[16] Adeyi A.A., Abayomi T.G., Purkait M.K., Mondal P. (2019). Adsorptive Removal of Phosphate from Aqueous Solution by Magnetic-Supported Kaolinite: Characteristics, Isotherm and Kinetic Studies. Open J. Appl. Sci..

[17] Rahman Q.I., Ahmad M., Misra S.K., Lohani M. (2013). Effective photocatalytic degradation of Rhodamine B dye by ZnO nanoparticles. Mater. Lett..

[18] Bhargava S.K., Tardio J., Prasad J., Fo K., Akolekar D.B., Grocott S.C. (2006). Wet oxidation and catalytic wet oxidation. Ind. Eng. Chem. Res..

[19] Bansal J., Hafiz A.K., Sharma S.N. (2020). Photoreduction of dye with noble metal gold permeated with metal oxide titania. J. Nanosci. Nanotechnol..

[20] Gupta V.K., Sharma G., Pathania D., Kothiyal N.C. (2015). Nanocomposite pectin Zr(IV) selenotungstophosphate for adsorptional/photocatalytic remediation of methylene blue and malachite green dyes from aqueous system. J. Ind. Eng. Chem..

[21] Tabbabi K., Karmous T. (2018). Use of lichens as bio-indicators in assessing the level of air pollution in the region of Bizerte Study of pollutants attached to frond lichens by atomic spectroscopy and emission spectroscopy by plasma torch. Mor. J. Chem..

[22] Kakavandi B., Takdastan A., Pourfadakari S., Ahmadmoazzam M., Jorfi S. (2019). Heterogeneous catalytic degradation of organic compounds using nanoscale zero-valent iron supported on kaolinite: Mechanism, kinetic and feasibility studies. J. Taiwan Inst. Chem. Eng..

[23] Diawara M., Kamissoko M., Rahali S., Samaké D., Tamboura M., Diawara B., Seydou M. (2021). A Computational Exploration of Ammonia Adsorption on the Kaolinite Clay Surface. Chem. Afr..

[24] Tahir H., Saud A., Saad M. (2018). Synthesis of kaolin loaded Ag and Ni nanocomposites and their applicability for the removal of malachite green oxalate dye. Iran. J. Chem. Chem. Eng..

[25] Hashemian S., Shahedi M.R. (2013). Novel Ag/Kaolin Nanocomposite as Adsorbent for Removal of Acid Cyanine 5R from Aqueous Solution. J. Chem..

[26] Assila O., Miyah Y., Nahali L., Elbadraoui A., Nenov V., Elkhazzan B., Zerrouq F., Kherbeche A. (2021). Copper-impregnation on natural material as promising catalysts for the wet hydrogen peroxide catalytic oxidation of methyl Green. Mor. J. Chem..

[27] Ghoniem M.G., Ali F.A.M., Abdulkhair B.Y., Elamin M.R.A., Alqahtani A.M., Rahali S., Ben Aissa M.A. (2022). Highly Selective Removal of Cationic Dyes from Wastewater by MgO Nanorods. Nanomaterials.

[28] Shuang S., Zhang Z. (2018). The Effect of Annealing Treatment and Atom Layer Deposition to Au/Pt Nanoparticles-Decorated TiO_2_ Nanorods as Photocatalysts. Molecules.

[29] Ali F.A.M., Ghoniem M.G., Diawara M., Rahali S., Abdulkhair B.Y., Elamin M.R., Ben Aissa M.A., Seydou M. (2022). Enhanced adsorptive removal of indigo carmine dye by bismuth oxide doped MgO based adsorbents from aqueous solution: Equilibrium, kinetic and computational studies. RSC Adv..

[30] Abdulkhair B., Salih M., Modwi A., Adam F., Elamin N., Seydou M., Rahali S. (2021). Adsorption behavior of barium ions onto ZnO surfaces: Experiments associated with DFT calculations. J. Mol. Struct..

[31] Lanfredi S., Nobre M.A.L., Poon P.S., Matos J. (2020). Hybrid Material Based on an Amorphous-Carbon Matrix and ZnO/Zn for the Solar Photocatalytic Degradation of Basic Blue 41. Molecules.

[32] Rahali S., Ali Ben Aissa M., Khezami L., Elamin N., Seydou M., Moidwi A. (2021). Adsorption Behavior of Congo Red onto Barium-Doped ZnO Nanoparticles: Correlation between Experi-mental Results and DFT Calculations. Langmuir.

[33] Wang S., Gao S., Tian J., Wang Q., Wang T., Hao X., Cui F. (2020). A stable and easily prepared copper oxide catalyst for degradation of organic pollutants by peroxymonosulfate activation. J. Hazard. Mater..

[34] Rezvani M.A., Miri O.F. (2019). Synthesis and characterization of PWMn/NiO/PAN nanosphere composite with superior catalytic activity for oxidative desulfurization of real fuel. Chem. Eng. J..

[35] Hadjltaief H.B., Ben Ameur S., Da Costa P., Ben Zina M., Galvez M.E. (2018). Photocatalytic decolorization of cationic and anionic dyes over ZnO nanoparticle immobilized on natural Tunisian clay. Appl. Clay Sci..

[36] El-Kemary M., Nagy N., El-Mehasseb I. (2013). Nickel oxide nanoparticles: Synthesis and spectral studies of interactions with glucose. Mater. Sci. Semicond. Process..

[37] Jiang Y., Li X., Qin Z., Ji H. (2016). Preparation of Ni/bentonite catalyst and its applications in the catalytic hydrogenation of nitrobenzene to aniline. Chin. J. Chem. Eng..

[38] Teimouri M., Khorsandi H., Aghapour A.A., Jafari S.J. (2018). Degradation and Mineralization of Malachite Green Dye in Aqueous Solution by Electro-Fenton Process Using Iron Electrodes. Int. J. Health Life Sci..

[39] Ayodele O.B., Lim J.K., Hameed B.H. (2012). Degradation of phenol in photo-Fenton process by phosphoric acid modified kaolin supported ferric-oxalate catalyst: Optimization and kinetic modeling. Chem. Eng. J..

[40] Karimi L., Zohoori S., Yazdanshenas M.E. (2014). Photocatalytic degradation of azo dyes in aqueous solutions under UV irradiation using nano-strontium titanate as the nanophotocatalyst. J. Saudi Chem. Soc..

[41] Wang N.N., Hu Q., Hao L.L., Zhao Q. (2019). Degradation of Acid Organic 7 by Modified Coal Fly Ash-Catalyzed Fenton-like Process: Kinetics and Mechanism Study. Int. J. Environ. Sci. Technol..

[42] Zhan Y., Zhou X., Fu B., Chen Y. (2011). Catalytic wet peroxide oxidation of azo dye (Direct Blue 15) using solvothermally synthesized copper hydroxide nitrate as catalyst. J. Hazard. Mater..

[43] Barka N., Qourzal S., Assabbane A., Nounah A., Ait-Ichou Y. (2013). Photocatalytic degradation of an azo reactive dye, Reactive Yellow 84, in water using an industrial titanium dioxide coated media. Arab. J. Chem..

[44] Ertugay N., Acar F.N. (2013). Removal of COD and color from direct blue 71 azo dye wastewater by Fenton’s oxidation: Kinetic study. Arab. J. Chem..

[45] Li X., Xiong Z., Ruan X., Xia D., Zeng Q., Xu A. (2012). Kinetics and mechanism of organic pollutants degradation with cobalt–bicarbonate–hydrogen peroxide system: Investigation of the role of substrates. Appl. Catal. A.

[46] Lucas M.S., Peres J.A. (2009). Removal of COD from olive mill wastewater by Fenton’s reagent: Kinetic study. J. Hazard. Mater..

[47] Behnajady M.A., Modirshahla N., Shokri M., Vahid B. (2008). Effect of operational parameters on degradation of Malachite Green by ultrasonic irradiation. Ultrason. Sonochem..

[48] Bencedira S., Bechiri O. (2020). The oxidation study of fuchsine acid dye by HP_2_W_15_Mo_3_Co_2.5_O_62_,20H_2_O/H_2_O_2_: Temperature effect, kinetic and thermodynamic study. Chem. Data Collect..

[49] Dang G.H., Le T.T.A., Ta A.K., Ho T.N.T., Pham T.V., Doan T.V.H., Luong T.H.V. (2020). Removal of Congo red and malachite green from aqueous solution using heterogeneous Ag/ZnCo-ZIF catalyst in the presence of hydrogen peroxide. Green Process. Synth..

[50] Milenova K., Zaharieva K., Avramova I., Stambolova I., Blaskov V., Dimitrov L., Eliyas A. (2016). CoO/Al_2_O_3_, CuO/Al_2_O_3_ and NiO/Al_2_O_3_ catalysts for photodegradation of malachite green dye under UV-irradiation. Int. J. Sci. Tech. Innov. Ind. MTM.

[51] Saad A.M., Abukhadra M.R., Abdel-Kader Ahmed S., Elzanaty A.M., Mady A.H., Betiha M.A., Shim J.-J., Rabie A.M. (2020). Photocatalytic degradation of malachite green dye using chitosan supported ZnO and Ce-ZnO nano-flowers under visible light. J. Environ. Manag..

[52] Mohamed R.M., Shawky A. (2018). CNT supported Mn-doped ZnO nanoparticles: Simple synthesis and improved photocatalytic activity for degradation of malachite green dye under visible light. Appl. Nanosci..

